# Evolution from KRAS G12C-mutant to ALK fusion-positive stage IV lung adenocarcinoma following immune checkpoint inhibitor therapy: a case report

**DOI:** 10.3389/fonc.2026.1929194

**Published:** 2026-08-12

**Authors:** Feng Xu, Hongli Pu, Na Yan, Yunhui Wei

**Affiliations:** 1 Department of Oncology and Hematology, Xiangyang No.6 People’s Hospital, Xiangyang, Hubei, China; 2 Wuhan Dian Medical Laboratory Co., Ltd., Wuhan, Hubei, China; 3 Key Laboratory of Digital Technology in Medical Diagnostics of Zhejiang Province, Dian Diagnostics Group Co. Ltd., Hangzhou, Zhejiang, China

**Keywords:** acquired resistance, clonal evolution, EML4-ALK fusion, immune checkpoint inhibitor, KRAS G12C, lung adenocarcinoma

## Abstract

**Background:**

Immune checkpoint inhibitors (ICIs) have become standard treatment for KRAS-mutant lung adenocarcinoma, but acquired resistance inevitably develops. Tumor clonal evolution under therapeutic pressure is a key resistance mechanism. The emergence of a new oncogenic driver mutually exclusive with the original one is rare.

**Case report:**

We report a 51-year-old female with stage IV lung adenocarcinoma harboring a KRAS G12C mutation along with TP53 and RB1 alterations. She received first-line therapy including the ICI tislelizumab in combination with chemotherapy and bevacizumab, achieving a partial response (PR) that was maintained for approximately 12 months (from April 2024 to April 2025), before subsequent disease progression was documented 26 months after treatment initiation. Upon disease progression, a repeat biopsy of the lung lesion revealed persistent adenocarcinoma histology without small-cell or squamous transformation. Remarkably, next-generation sequencing demonstrated the loss of the original KRAS G12C mutation and the emergence of a novel EML4-ALK fusion. The patient was switched to the ALK inhibitor ensartinib, achieving a second PR.

**Conclusion:**

This case provides supporting evidence consistent with the selection of a pre-existing ALK fusion clone in a KRAS-mutant lung adenocarcinoma under chemo-immunotherapy pressure. It also highlights the clinical value of re-biopsy at progression to guide subsequent targeted therapy.

## Introduction

Lung adenocarcinoma (LUAD) is the predominant histological subtype of non-small cell lung cancer (NSCLC) (1). Mutations in KRAS, especially the KRAS G12C variant, represent one of the most common driver alterations in this population. The KRAS G12C mutation accounts for 10%–13% of NSCLC cases in Western populations and 8%–10% among Chinese NSCLC patients, and is predominantly detected in smokers, a population with an overall poor prognosis (2). The combination of immune checkpoint inhibitors (ICIs) and platinum-based chemotherapy remains the standard first-line regimen for advanced KRAS G12C-mutated LUAD. Despite initial clinical benefits, most patients eventually develop acquired resistance to ICI-based therapy, which severely limits long-term survival outcomes (3, 4).

Acquired resistance to PD-(L)1 blockade in NSCLC is characterized by highly heterogeneous mechanisms, involving dynamic tumor clonal evolution, immune microenvironment remodeling, and the emergence of novel oncogenic driver alterations (1, 5). Recent large-scale paired-sample analyses have suggested that prolonged ICI treatment exerts strong selective pressure on tumor cell populations, eliminating immune-sensitive dominant clones and enabling the outgrowth of pre-existing subclones with intrinsic immune evasion capacity. Genomic instability under immunotherapy further drives the activation of alternative oncogenic pathways, serving as a critical cause of treatment failure (6, 7).

In NSCLC, KRAS mutations and anaplastic lymphoma kinase (ALK) rearrangements are well-recognized mutually exclusive driver events in treatment-naive patients (8, 9). Their co-occurrence or sequential transformation during treatment is extremely rare, owing to overlapping downstream signaling (6)pathways that make dual-driven tumorigenesis biologically unfavorable. Only sporadic synchronous cases have been reported in previous studies, while sequential clonal switching from KRAS G12C mutation to EML4-ALK fusion under ICI pressure has rarely been documented (10–12).

Herein, we report a rare clinical case of advanced KRAS G12C-mutated lung adenocarcinoma that progressed after long-term ICI plus chemotherapy treatment. Comprehensive genomic profiling confirmed that the tumor evolved to EML4-ALK fusion positivity, and the patient achieved favorable disease control after subsequent salvage therapy with ALK tyrosine kinase inhibitor (TKI). This case provides a clinical observation compatible with clonal evolution as a potential mechanism of ICI resistance, and highlights the clinical value of dynamic genomic monitoring for patients experiencing immunotherapy failure.

## Case presentation

**Figure 1:**
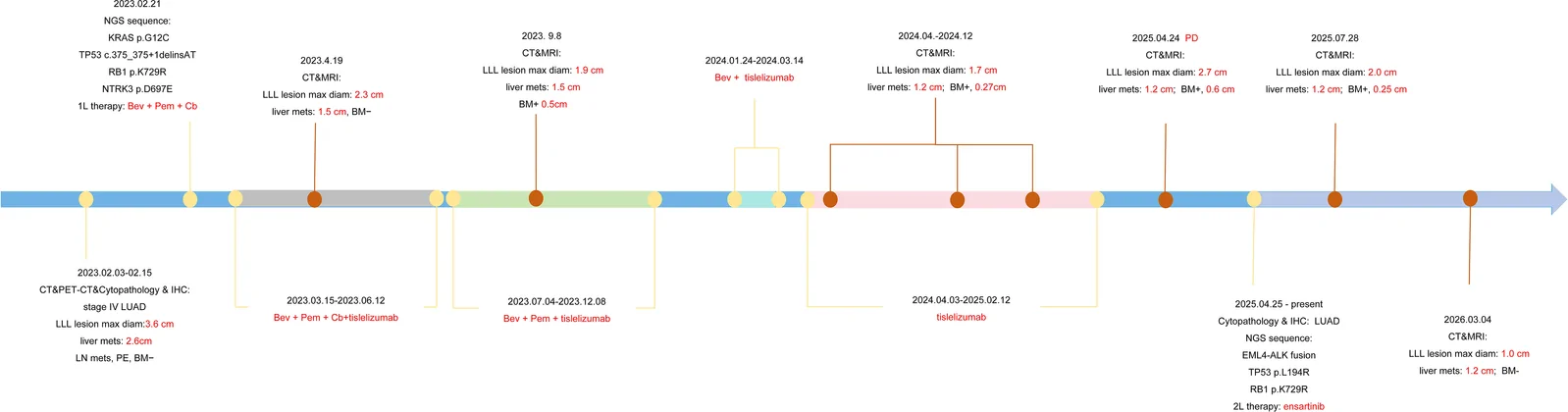
Diagnostic and therapeutic timeline of the patient with stage IV lung adenocarcinoma. Timeline illustrating the patient’s clinical course, diagnostic workup, genetic alterations, treatment regimens, and radiographic responses from initial diagnosis (February 2023) to the last follow-up (March 2026). Key molecular findings, including the initial KRAS G12C mutation and the emergent EML4-ALK fusion detected at progression, are shown along with corresponding therapeutic interventions and best responses. LUAD, lung adenocarcinoma; LLL, left lower lobe; LN, lymph node; PE, pleural effusion; BM, brain metastasis; Bev, bevacizumab; Pem, pemetrexed; Cb, carboplatin; ICI, immune checkpoint inhibitor; 1L, first-line; 2L, second-line; NGS, next-generation sequencing; PR, partial response; SD, stable disease; PD, progressive disease.

**Figure 2:**
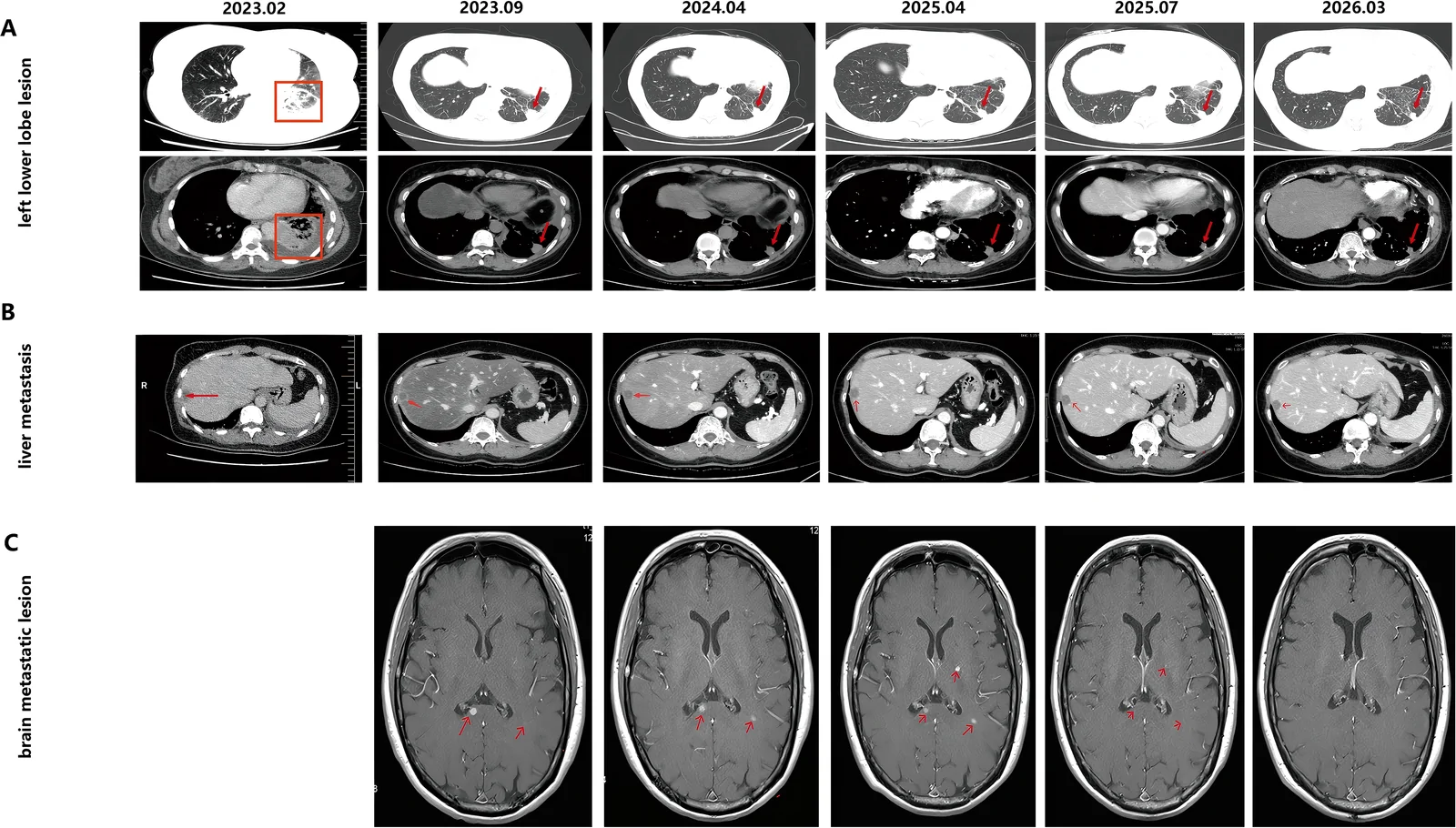
Serial radiological imaging monitoring of target lesions across all disease sites from 2023 to 2026. **(A)** Left lower lobe primary lesion: Upper panels represent lung window CT scans; lower panels show mediastinal window CT scans, with red arrows marking the primary tumor. **(B)** Hepatic metastases: Abdominal CT slices with red arrows indicating liver metastatic lesions, which remained stable throughout ensartinib treatment. **(C)** Intracranial metastases: Brain MRI sequences displaying cerebral metastatic foci (red arrows), which achieved complete radiographic resolution by March 2026 follow-up.

On February 3, 2023, a 51-year-old non-smoking woman presented with progressive dyspnea and chest tightness, and a comprehensive diagnostic workup was subsequently performed. A comprehensive timeline of the patient’s clinical course is presented in **Figure 1**. Contrast-enhanced computed tomography (CT) of the chest revealed an infectious lesion in the left lower lobe, mediastinal lymphadenopathy, and left pleural effusion (**Figure 2**). Subsequently, whole-body ¹^8^F-FDG PET/CT (February 15, 2023) demonstrated a hypermetabolic soft tissue mass in the left lower lobe (3.6×3.0×2.8 cm, SUVmax 16.1), along with multiple hypermetabolic lesions in the mediastinal and left hilar lymph nodes (largest 2.7×1.5 cm, SUVmax 11.9), and hypodense liver nodules with abnormal increase in glucose metabolism (largest 2.6×2.2 cm, SUVmax 6.8), consistent with stage IV lung cancer. Additionally, a left-sided pleural effusion and a soft tissue density along the posterior mediastinum (2.1×0.8 cm, SUVmax 11.7), suggesting metastatic lymphadenopathy, were observed. A small patchy slightly high-density lesion adjacent to the posterior horn of the left lateral ventricle showed no elevated FDG metabolism; regular follow-up was recommended. Cytological examination of the pleural effusion revealed malignant cells. Subsequent immunohistochemical (IHC) analysis of the cell block demonstrated positive immunoreactivity for CK7, Cam5.2, TTF-1, and Napsin-A, while being negative for CK5/6, CK20, PAX8, CDX2, and calretinin. These findings confirmed the diagnosis of primary lung adenocarcinoma. Baseline molecular profiling was performed on a pleural effusion cell-block specimen obtained in March 2023 using the Di’an Basic Lung Cancer Targeted Therapy Gene Detection panel on the Illumina NextSeq platform with a hybridization capture-based approach. The panel covered 16 cancer-related genes (ALK, BRAF, EGFR, ERBB2, KRAS, MET, PIK3CA, ROS1, RET, NRAS, PTEN, RB1, TP53, NTRK1, NTRK2, and NTRK3) and was designed to detect SNVs, InDels, CNVs, and gene fusions, with a validated limit of detection of 2% for SNVs/InDels and 5 supporting reads for gene fusions. The reference genome was UCSC hg19 (Feb. 2009). The baseline NGS assay (mean depth 3,127.8×, tumor fraction 5%) identified KRAS p.G12C (c.34G>T, mutation abundance: 5.62%), TP53 c.375_375 + 1delinsAT (mutation abundance: 5.06%), RB1 p.K729R (c.2186A>G, mutation abundance: 36.97%), and NTRK3 p.D697E (c.2091C>A, mutation abundance: 1.53%) (**Supplementary Table 1**; **Supplementary Figure 1A**). PD-L1 expression was not assessed at baseline due to insufficient cellularity of the pleural effusion cell-block specimen. At the time of diagnosis in February 2023, and as of the submission of this report, no targeted therapy had been approved in China for the first-line treatment of KRAS G12C-mutant lung adenocarcinoma. Based on the patient’s molecular profile, particularly the presence of KRAS G12C mutation and concurrent genetic alterations, and in accordance with contemporary clinical practice guidelines, immune checkpoint inhibitor (ICI) combined with platinum-based chemotherapy was recommended as the standard first-line treatment regimen for this molecular subtype of advanced lung adenocarcinoma.

First-line systemic therapy was initiated on February 21, 2023, with bevacizumab (750 mg, day 0), pemetrexed (700 mg), and carboplatin (600 mg) (Bev+CP regimen), given every 21 days. Starting from the second cycle (March 15, 2023), the ICI tislelizumab (200 mg, day 1) was added. The patient received a total of six cycles of this quadruple regimen (bevacizumab + tislelizumab + pemetrexed + carboplatin). From July to December 2023, maintenance therapy was continued with tislelizumab (200 mg), bevacizumab (750 mg), and pemetrexed (700 mg), administered every 21 days. From January to February 2024, the maintenance regimen was adjusted to bevacizumab combined with tislelizumab. In February 2024, the patient developed grade 2 hand stiffness, which was considered related to bevacizumab, so bevacizumab was discontinued. Starting from April 2024, the patient continued tislelizumab monotherapy (200 mg every 21 days).

From February 2023 (baseline assessment) to April 2024, the patient received regular follow-up and first-line systemic therapy. The longest diameter of the target lesion in the left lower lobe was 3.6 cm at baseline and decreased to 2.3 cm after 3 cycles of treatment (April 23, 2023), showing a marked response trend (36.1% reduction from baseline). During subsequent follow-up, the lesion exhibited continuous and gradual shrinkage, and further decreased to 1.5 cm on April 24, 2024, representing a 58.3% reduction from baseline, confirming a partial response (PR) per RECIST 1.1 criteria. The confirmed PR status was maintained from April 2024 until April 2025, yielding a duration of response of approximately 12 months before progression was documented. Thereafter until December 2024, the lesion size remained stable, and the therapeutic response was assessed as stable disease (SD).

Liver metastases showed a similar response. The largest liver lesion measured 2.6 cm at baseline, decreased to 1.5 cm on September 8, 2023, and further reduced to 1.2 cm by April 2024, remaining stable at that size thereafter. Intracranial lesions were first detected on contrast-enhanced brain MRI in September 2023 (7 months after treatment initiation), with the largest lesion measuring 0.5 cm. Notably, these lesions were absent at baseline. Serial brain MRI follow-up demonstrated gradual regression of these lesions without any local intervention, with the largest lesion decreasing to 0.27 cm by April 2024. The patient remained completely asymptomatic throughout this period. Per iRECIST criteria, the appearance of new lesions on first follow-up is categorized as iUPD (immune unconfirmed progressive disease), requiring confirmatory imaging to distinguish true progression from pseudoprogression. In this patient, the subsequent regression of these new lesions, together with the ongoing systemic partial response and absence of neurological symptoms, supported the retrospective interpretation of these findings as immune-related pseudoprogression (iCPD) rather than true intracranial progression. The patient declined local radiotherapy; the decision to continue systemic therapy was based on the above imaging trajectory and clinical stability.

On April 23, 2025 (26 months after initial treatment), imaging evaluation indicated progressive disease (PD). The maximum diameter of the left lower lobe lesion increased to 2.7 cm. Progressive changes were also observed in some metastatic lesions: the largest liver lesion remained stable at 1.2 cm, but the largest intracranial lesion increased to 0.6 cm.

**Figure 3:**
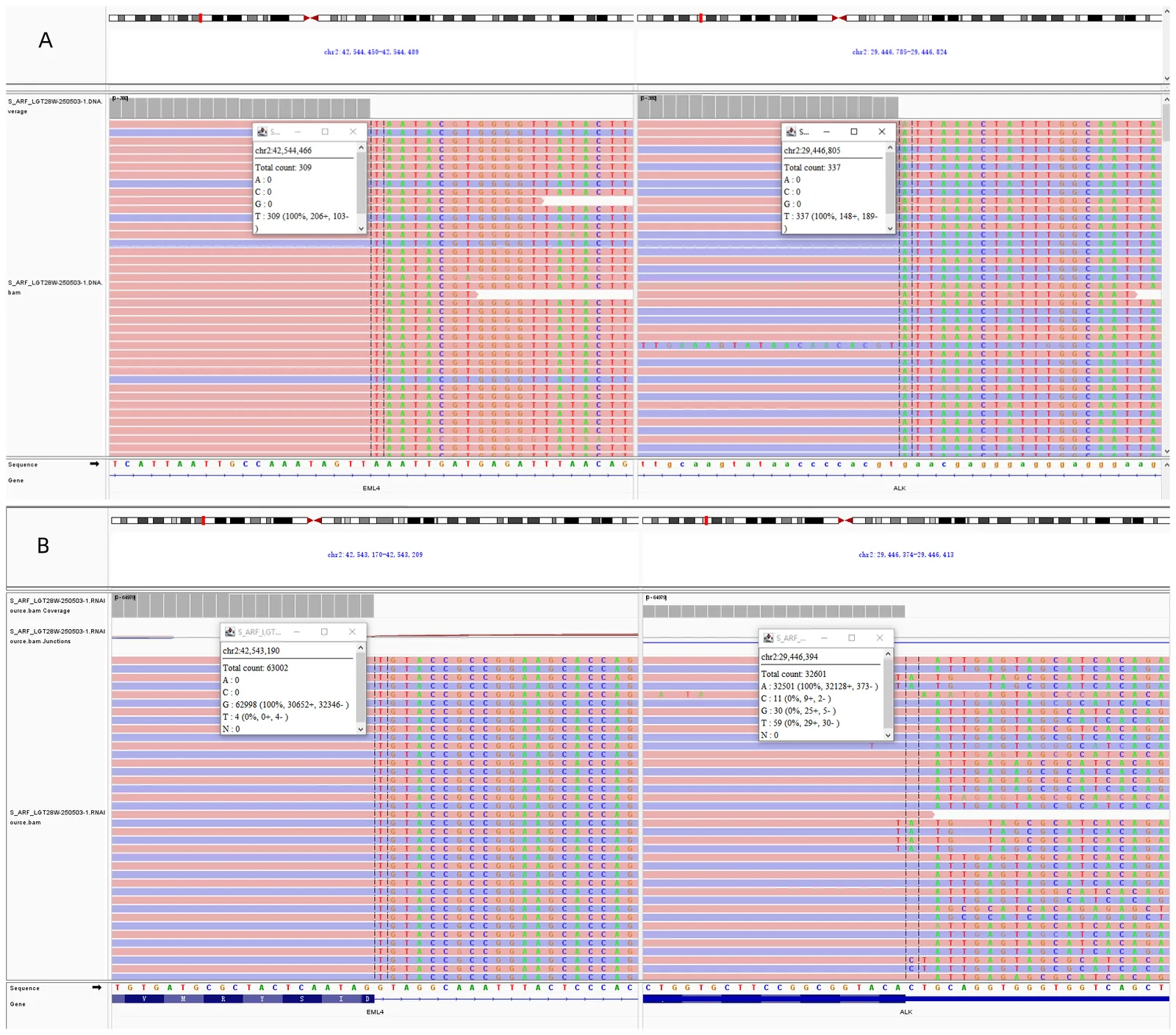
Molecular evidence of the emergent EML4-ALK fusion in the progression biopsy (May 2025, core needle biopsy of lung lesion). **(A)** DNA-based NGS IGV snapshot showing split-read and discordant read-pair alignments spanning the EML4 exon 18 and ALK exon 20 breakpoint (E18:A20, V5’ variant) **(B)** RNA-based NGS IGV snapshot showing junction reads spanning the EML4-ALK fusion transcript. **Supplementary Table 1**. Comparison of sequencing assay parameters and quality metrics between baseline and progression specimens. **Supplementary Figure 1**. IGV snapshots demonstrating negative findings for the mutually exclusive driver alterations at both time points. **(A)** Baseline specimen (March 2023, pleural effusion cell block). IGV view of the ALK genomic locus showing no evidence of ALK rearrangement. No discordant read pairs, split-reads, or junction reads supporting any ALK fusion were detected at the ALK breakpoint regions or its major fusion partners including EML4, even upon manual review of unfiltered BAM files with the threshold lowered to 2 supporting reads. **(B)** Progression specimen (May 2025, lung core needle biopsy). IGV view of the KRAS G12C hotspot showing no residual detectable reads for the mutant allele, indicating loss of detectable KRAS G12C mutation in this specimen at progression.

A repeat biopsy of the progressive left lung mass was performed on April 24, 2025. Histopathological examination confirmed lung adenocarcinoma (predominantly solid pattern). IHC was positive for CK7, TTF-1, Napsin-A, and EGFR (the latter included as part of the routine immunohistochemistry panel to support adenocarcinoma lineage; this does not substitute for EGFR mutation testing, which was performed by NGS), and negative for p40, CgA, Syn, and CD56, ruling out squamous or small-cell transformation. The Ki-67 proliferation index was 10%. P53 showed wild-type expression pattern (positive, scattered). At disease progression (May 2025), a core needle biopsy of the lung lesion was analyzed using the Lung Cancer Precision Diagnosis panel on the same Illumina platform with parallel DNA and RNA workflows. Crucially, the repeat NGS analysis (DNA mean depth 3,658.67×, RNA 2.2 million reads, tumor fraction 70%) detected an EML4-ALK fusion (E18:A20, V5′) at DNA VAF 21.16% and RNA supporting reads of 32,103, with no residual KRAS G12C mutation. (**Figure 3**; **Supplementary Table 1**; **Supplementary Figure 1B**). Interestingly, the TP53 mutation profile had changed: the original TP53 c.375_375 + 1delinsAT was no longer detected, but a novel TP53 p.L194R missense mutation emerged (mutation abundance: 5.26%). The RB1 p.K729R mutation persisted (mutation abundance: 46.96%). Based on this molecular evolution, the ICI was discontinued, and the patient was started on the ALK tyrosine kinase inhibitor ensartinib (225 mg once daily) in early May 2025.

After switching to ensartinib, restaging imaging performed in July 2025 demonstrated marked regression of the primary left lower lobe lesion, which decreased from 2.7 cm to 2.0 cm (a 26% reduction), along with substantial improvement of intracranial metastases. The dominant intracranial lesion shrank from 0.6 cm to 0.25 cm, and some small brain metastases became nearly undetectable on follow-up imaging. Subsequent follow-up in November 2025 showed continued shrinkage of pulmonary lesions and further regression of cerebral metastatic foci. At the last follow-up in March 2026, imaging revealed sustained tumor response: the primary left lower lobe lesion had further decreased to 1.0 cm (a 63% reduction from the 2.7 cm baseline at ensartinib initiation), hepatic metastatic lesions remained stable at 1.2 cm, and all intracranial metastases achieved complete radiographic resolution with no residual lesions identified. No new metastatic lesions or signs of disease progression were observed during ensartinib treatment. According to RECIST 1.1 criteria, the best overall response during ensartinib treatment was partial response (PR).

## Discussion

This case report presents an rare clonal evolutionary event in advanced lung adenocarcinoma (LUAD): a tumor initially driven by the *KRAS* G12C mutation progressed to harbor EML4-ALK fusion after long-term treatment with immune checkpoint inhibitors (ICIs) combined with chemotherapy. Accumulated large-scale genomic analyses have validated that *KRAS* mutations and *ALK* rearrangements are classic mutually exclusive driver alterations in treatment-naive non-small cell lung cancer (NSCLC) cohorts, and concurrent or sequential transformation of these two oncogenic drivers is exceedingly rare in routine clinical practice ASCO Publications (8). Such mutual exclusivity is biologically attributed to overlapping downstream RAS/RAF/MEK signaling cascades, which makes dual-driven tumorigenesis genetically unfavorable. Different from the few previously reported cases of synchronous coexistence of KRAS mutation and ALK fusion (10, 11), fusion, this case demonstrates a clonal evolutionary pattern consistent with selection pressure from chemo-immunotherapy, although the contributory role of ICI therapy cannot be definitively established. Combined with the findings from Ricciuti et al. (6), this phenomenon offers an illustrative example of potential genomic evolution under treatment pressure and expands the clinical spectrum of secondary driver gene emergence in KRAS-mutant LUAD.

The persistent RB1 alteration (VAF 37-47% at both time points) provides a critical clue to the clonal architecture, strongly suggesting a common ancestral clone from which divergent subclones evolved. Under this branching model, one subclone acquired KRAS G12C with concurrent TP53 inactivation, while a separate subclone harbored the EML4-ALK fusion, with the latter being selected for expansion under chemo-immunotherapy pressure. However, several technical caveats warrant consideration when interpreting this apparent clonal evolution. First, the baseline specimen was a pleural effusion cell block with low tumor cellularity (5%, compared to 70% in the progression tissue biopsy), which inherently reduces sensitivity for detecting low-abundance subclones and introduces potential sampling bias due to spatial heterogeneity between pleural and pulmonary sites. Cell-block preparations from pleural effusions are a practical and reliable alternative for molecular testing when tissue biopsy is not feasible and are recommended by current guidelines; however, they are subject to important limitations, including variable tumor cellularity, potential sampling bias, and reduced sensitivity—particularly when tumor cell proportion falls below 10%. Second, although the baseline NGS panel was technically capable of detecting ALK fusions (with comprehensive coverage of ALK and EML4 breakpoints) and manual BAM file review revealed zero ALK-supporting reads, we cannot definitively exclude the presence of a minute ALK-positive clone below the assay’s limit of detection. Third, the substantially higher tumor content (70%) and parallel DNA/RNA workflow at progression may have facilitated detection of a previously undetectable subclone. Furthermore, the baseline assay was DNA-based only, whereas the progression assay employed a combined DNA and RNA workflow, which may have enhanced fusion detection sensitivity at the transcript level. We also acknowledge that orthogonal validation of the ALK rearrangement by IHC, FISH, or RT-PCR was not feasible due to exhaustion of the limited core needle biopsy material after histopathological evaluation and NGS testing—an inherent limitation of small biopsy specimens in the real-world clinical setting. Nevertheless, the concurrent detection of the EML4-ALK fusion by independent DNA and RNA NGS workflows, with high RNA junction read support (32,103 reads), substantially strengthens the molecular evidence. Collectively, these findings are most consistent with clonal selection and expansion of a pre-existing dormant ALK-rearranged subclone under treatment pressure, rather than definitive proof of *de novo* fusion acquisition. We acknowledge that definitive phylogenetic reconstruction would require multi-region or longitudinal sampling with higher-density molecular tracking, which was not feasible in this clinical setting. This interpretation aligns with the broader literature on branching clonal evolution as a mechanism of acquired resistance to targeted and immunotherapies (13). Finally, we acknowledge that the initial and progression biopsies were obtained from different anatomical sites (pleural effusion vs. lung parenchyma), and the observed molecular changes may therefore reflect spatial heterogeneity rather than, or in addition to, temporal clonal evolution. This limitation is inherent to the clinical reality of repeat biopsy but should be considered when interpreting the findings. Recent work has explored percutaneous closed pleural brushing combined with cell block technique as an alternative approach to improve diagnostic yield in malignant pleural effusion (14). These considerations underscore the importance of interpreting molecular findings from cytology specimens with caution and, whenever possible, obtaining tissue biopsy for confirmation at progression.

This unique clinical scenario delivers critical practical implications for the standardized management of advanced NSCLC after ICI failure. In clinical practice, disease progression after long-term disease control with ICI regimens in *KRAS*-mutant LUAD is conventionally attributed to non-specific immune resistance or adaptive immune tolerance, and clinicians often adjust treatment regimens empirically without repeated molecular testing. However, as highlighted by multi-omics analyses of ICI resistance, genomic evolution and secondary driver alterations are common events during immunotherapy failure, and these changes may not be reliably identified by imaging alone. Our case fully demonstrates the clinical necessity of repeat tumor biopsy and dynamic comprehensive genomic profiling at the time of disease progression, regardless of the initial driver gene status. For patients experiencing progression after sustained ICI efficacy, clinicians should actively screen for secondary targetable driver genes rather than simply attributing progression to non-targeted immune resistance. Timely identification of the newly emerged EML4-ALK fusion and subsequent administration of ALK tyrosine kinase inhibitor (TKI) salvage therapy achieved favorable disease control in this patient, which proves that dynamic genomic monitoring is the key to identifying potential targeted therapeutic opportunities and breaking through ICI resistance.

Notably, this case also verifies that molecular evolution can occur independently of histological transformation under ICI selective pressure. Throughout the entire treatment and disease progression course, the histological type of the tumor remained stable as lung adenocarcinoma, without transformation to small cell lung cancer or squamous cell carcinoma, a common histological transformation pattern observed in targeted therapy resistance.

In conclusion, this rare case provides a clinical observation compatible with clonal evolution from KRAS G12C to EML4-ALK fusion in lung adenocarcinoma under chemo-immunotherapy pressure, although the precise role of ICI therapy in this process remains speculative. Consistent with the broader literature, this case supports the concept that tumor clonal evolution and genomic heterogeneity may contribute to acquired resistance to PD-(L)1 blockade. However, spatial heterogeneity between biopsy sites and differences in tumor cellularity and assay sensitivity represent important alternative explanations that cannot be excluded. We strongly recommend routine repeat biopsy and dynamic genomic testing for all NSCLC patients with disease progression after immunotherapy, to timely identify secondary actionable driver genes and maximize the benefits of precision targeted therapy.

## Data Availability

The original contributions presented in the study are included in the article/**Supplementary Material**. Further inquiries can be directed to the corresponding author.

## Supplementary material

Supplementary Figure 1 IGV snapshots demonstrating negative findings for the mutually exclusive driver alterations at both time points. **(A)** Baseline specimen (March 2023, pleural effusion cell block). IGV view of the ALK genomic locus showing no evidence of ALK rearrangement. No discordant read pairs, split-reads, or junction reads supporting any ALK fusion were detected at the ALK breakpoint regions or its major fusion partners including EML4, even upon manual review of unfiltered BAM files with the threshold lowered to 2 supporting reads. **(B)** Progression specimen (May 2025, lung core needle biopsy). IGV view of the KRAS G12C hotspot showing no residual detectable reads for the mutant allele, indicating loss of detectable KRAS G12C mutation in this specimen at progression.
